# Integrating care for high-risk patients in England using the virtual ward model: lessons in the process of care integration from three case sites

**DOI:** 10.5334/ijic.1150

**Published:** 2013-11-05

**Authors:** Geraint Lewis, Rhema Vaithianathan, Lorraine Wright, Mary R Brice, Paul Lovell, Seth Rankin, Martin Bardsley

**Affiliations:** NHS England, Quarry House, Leeds, UK; Economics, University of Auckland, Owen G Glenn Building, 12 Grafton Road, Auckland, New Zealand; Singapore Management University, Singapore; Nursing and Quality, NHS Blood and Transplant, Oak House, Reeds Crescent, Watford, Hertfordshire, UK; Heart Failure Palliative Care Nurse Consultant, St Christopher's Hospice, 51-59 Lawrie Park Road, Sydenham, UK; East Street Surgery, South Molton, Devon, UK; Wandsworth Medical Centre, 90-92 Garratt Lane, London, UK; Research, The Nuffield Trust, 59 New Cavendish Street, London, UK

**Keywords:** patient readmission, hospitalisation, algorithms, integrated delivery of health care, patient care team, preventive health services

## Abstract

**Background:**

Patients at high risk of emergency hospitalisation are particularly likely to experience fragmentation in care. The virtual ward model attempts to integrate health and social care by offering multidisciplinary case management to people at high predicted risk of unplanned hospitalisation.

**Objective:**

To describe the care practice in three virtual ward sites in England and to explore how well each site had achieved meaningful integration.

**Method:**

Case studies conducted in Croydon, Devon and Wandsworth during 2011–2012, consisting of semi-structured interviews, workshops, and site visits.

**Results:**

Different versions of the virtual wards intervention had been implemented in each site. In Croydon, multidisciplinary care had reverted back to one-to-one case management.

**Conclusions:**

To integrate successfully, virtual ward projects should safeguard the multidisciplinary nature of the intervention, ensure the active involvement of General Practitioners, and establish feedback processes to monitor performance such as the number of professions represented at each team meeting.

## Introduction

Patients at high risk of emergency hospitalisation typically have multiple chronic conditions, which are often compounded by psychological and social issues.[1,2] As a result of these complex and interacting needs, high-risk patients are more likely to experience fragmented care but also to derive greater benefit from improvements in the integration of health and social care [3]. This series of case studies describes three adaptations of the ‘virtual wards’ model from England, a model which aims to improve the integration of health and social care for patients at high risk of future hospitalisation.

### Background

To be successful, any hospital avoidance programme must target patients who are both at high risk of a future unplanned hospitalisation and who are likely to respond to the proposed intervention [4]. Importantly, this response needs to be large enough to justify the economic costs of the preventive intervention [5]. Designing a well-targeted and cost-effective intervention can be challenging given the complexity of high-risk patients’ needs.

In an attempt to address these challenges, an intervention known as ‘virtual wards’ was introduced at Croydon primary care trust in 2005 (see Box 1 for a description of what we have called the ‘original model’ [6]) and later adopted by several other sites across England [7].

The virtual ward model consists of two fundamental components: (1) using a predictive model to identify individual patients in a population who are at high risk of future unplanned hospital admission; and (2) offering these people a period of intensive, multidisciplinary, case management at home using the systems, staffing and daily routines of a hospital ward [4].

### Problem statement

A survey published in 2012 suggested that virtual wards had mutated in several ways from the original model described in Box 1 [4].

In this paper, we explore how well each of three virtual ward sites has achieved meaningful integration, based on the ‘success factors’ identified by Rosen and Shaw [8]. These factors are (1) the environment in which the project operated; (2) the organisational culture, infrastructure and processes; (3) the existence of effective multidisciplinary teams; and (4) whether patients actively participated in the care planning process. In our discussion, we reflect on the degree to which integrated care was achieved in the three projects, draw out a number of lessons for policy-makers, and suggest some avenues for future research.

## Methods

Ethics approval for this study was obtained through the Integrated Research Application System (National Research Ethics Service reference number 10/H0806/31). Our case studies were conducted during the period 2011-2012 by means of semi-structured interviews, workshops, and site visits. We held semi-structured interviews with a range of staff in each of the three study sites, including General Practitioners, nurses, finance department staff, and social care workers. A total of 14 staff members were interviewed with four from Croydon and five each from Wandsworth and Devon. During the course of the study, we held three workshops at which representatives from all three sites were present. The purpose of these workshops was to share and sense-check our emerging observations on the similarities and differences between the sites.

## Description of the care practice

Table 1 provides an overview of the virtual ward projects in the three study sites. Further details are available elsewhere [9].

We begin with the Croydon case study, as it was the original model, followed by two other sites that subsequently implemented their own versions of virtual wards: Devon and Wandsworth.

### Croydon

When they were implemented initially, the virtual wards in Croydon closely followed the original virtual ward model [6]. However, from 2007 onwards, the intervention morphed into standard (i.e. one-to-one) case management delivered by a community matron with the support of an administrative assistant. Therefore, Croydon virtual ward patients ceased receiving multidisciplinary case management from this time onwards.

#### Operating environment

When the Croydon virtual wards were launched in 2005, the health care policy environment in England was broadly supportive of this type of intervention. At the time, both the National Health Service and local authorities were being encouraged by the Department of Health to reduce hospital admissions [10]. However, although the Department of Health was encouraging the use of predictive models for case finding [11], it was advocating traditional (i.e., one-to-one) case management by a community matron rather than case management by a multidisciplinary team [12].

While the wider environment was broadly conducive to the virtual ward project, within Croydon primary care trust itself there were some unsupportive elements. For example, frequent changes in the organisation's management structure were seen as being disruptive and destabilising.

#### Organisational culture, infrastructure and processes

The organisational culture at Croydon primary care trust was generally supportive leading the implementation, the project was given. Since staff working in the trust headquarters were leading the implementation of virtual ward, the project was given a relatively high priority. All three practice-based commissioning groups signalled their support, as did the Professional Executive Committee and the Local Medical Committee. However, this official support did not appear to extend fully to ‘frontline’ general practitioners.

In terms of the infrastructure of the project, members of the public health department at Croydon had the experience and capacity to run the *Combined Predictive Model* in order to generate lists of high-risk patients [13]. Indeed, the primary care trust had a long history of using general practice encounter data to improve patient care. However, no portal was made available to display the output of the predictive model directly to general practitioners; instead, lists of patients were sent to the community matrons via secure email.

Many of the processes needed to support integration in the pilot phase of the project were described in a *Memorandum of Understanding* between the primary care trust and one of the practice-based commissioning groups. These processes included, for example, procedures for information exchange (such as extracting clinical information for new patients out of the general practice electronic record and importing this information into the electronic medical record used by virtual ward staff); an alert system to highlight any virtual ward patients who attended a local accident and emergency department; processes for agreeing a joint care plan with the London Ambulance Service; and a regular ‘mortality and morbidity’ meeting, which was attended by staff from multiple organisations, where adverse events were discussed. Rather tellingly, however, no general practitioners attended these meetings.

Finally, the community matrons and other community health care providers used a common electronic medical record; however, this system was not available to general practitioners, nor to local hospital staff, mental health professionals or social workers. Although shared assessments and common standards were developed during the pilot phase, these resources were subsequently withdrawn. Instead, community matrons were required to rely on informal collaboration with general practitioners to develop management plans for each patient; these joint plans were not always documented (although guidance published in 2013 offered a new vision and model for District Nursing, with case management at its core [14]). More generally, the lack of standardised transactional data for community health services (a shortcoming that is only now being addressed by the Health and Social Care Information Centre [15]) meant that attendance at multidisciplinary ward rounds was not adequately monitored.

#### Multidisciplinary teams

As originally envisaged, one of the main purposes of the virtual wards was to streamline and coordinate care for patients who were receiving treatment from multiple professionals and clinical teams. Initially, multidisciplinary virtual ward team meetings were held regularly. Known as ‘ward rounds’, these meetings were attended by the community matrons, district nurses, physiotherapists, occupational therapists, social workers and other community clinicians although no general practitioners attended.

However, these meetings were discontinued once the pilot phase ended. Therefore, although initially the virtual wards in Croydon were multidisciplinary in nature, after the pilot phase, each virtual ward consisted only of a community matron and a ward clerk. In other words, they were no longer providing multidisciplinary case management but rather traditional (one-to-one) case management. One of the reasons why this change may have occurred is that some staff viewed coordination as being less ‘important’ than directly providing care.

#### Activated patients

Although some patients being cared for on the Croydon virtual wards were ‘activated’ (i.e., they had the ‘knowledge, skills and confidence essential to managing their own health and healthcare’) [16], for many others, the virtual ward played a more paternalistic role in delivering care.

### Devon

Compared with Croydon, the virtual ward in Devon was more firmly rooted in primary care, with a general practitioner championing the development and implementation of the entire project. In this case study, we focus on the virtual ward covering South Molton and Chulmleigh; however, a further 22 virtual wards have since been established across the county of Devon, which build on an existing infrastructure of complex care teams.

#### Operating environment

The virtual ward project in Devon was developed in response to two related policies: reducing unplanned hospital admissions and providing more care closer to home. The original pilot virtual ward in South Molton and Chulmleigh was a practice-based commissioning initiative, which was championed by local general practitioners rather than being a primary care trust-led project. Later, however, the rollout of the project across the county was coordinated by the primary care trust in response to the national *Quality, Innovation, Productivity and Prevention* policy [17]. Another issue affecting the operating environment in Devon related to contractual law. Since the different virtual wards were commissioned and delivered by different organisations, any material change in the specifications of a virtual ward contract might require a re-tendering process. This process was seen as a time consuming and costly, especially where it involved mandatory re-advertising in the *Official Journal of the European Union* (Table 2).

When the project was first launched, there were only two community health provider organisations operating in Devon: the primary care trust itself, which covered most of the county, and a vertically integrated acute trust covering North Devon. Vertically integrated trusts provide care for patients at different stages in the care pathway, in this case acute hospital care and community care [18]. The contracts with these organisations were relatively inexplicit, allowing changes to be made without re-tendering. However, over the course of the project, the ‘Transforming Community Services’ process [19], required primary care trusts to divest themselves of their community provider arms. This change led to a proliferation of community provider organisations, and made the negotiation of contracts more tortuous.

National Health Service finances also had a major impact on the virtual ward project in Devon. The vertically integrated trust, which held the block contract for community services in the north of the county, began facing financial difficulties over the course of the project. These problems led to suspicions by virtual ward staff that the trust would divert resources away from community services and into hospital departments. Moreover, the perverse incentive on hospitals to admit more patients under the Payment by Results system [20] was an additional cause of mistrust between primary care commissioners and the acute trust. Finally, these misgivings were compounded when the acute trust proposed closing all community hospitals, and imposed a recruitment freeze on community posts.

#### Organisational culture, infrastructure and processes

A culture of integration had been emerging in Devon for several years prior to the establishment of the virtual ward. For example, a policy of greater integration for high-risk patients had led to the creation of *complex care teams*, which were jointly funded by the National Health Service and by the local authority. Within these teams, the line-management structure was unified across different organisations but some other issues remained unresolved. For example, the community care teams used a social care information technology system for documenting care that was unsuitable for health professionals to record their notes. Each community care team found its own solutions to such issues; however, these workarounds came at a cost of inconsistency across the county.

As in Croydon, one of the major challenges facing the project was to avoid a regression away from multidisciplinary case management back to traditional care. To prevent such a reversion from occurring, virtual ward and primary care staff were repeatedly reminded by the project champion that the funding for the project (designated as a *Local Enhanced Service*) was contingent on their providing proactive, multidisciplinary care. The virtual ward staff and general practice were also set targets, for example by specifying a minimum proportion of virtual ward patients that were to be identified by the predictive model as opposed to clinical referral.

One of the most contentious issues affecting the project in Devon was information governance, especially (1) the extraction of data from general practice systems for predictive modelling, and (2) the system for informing general practitioners about their patients’ predictive risk scores. In particular, it was difficult at times to navigate between the provisions of the Data Protection Act and the advice issued by various authorities, such as the National Information Governance Board, the NHS Care Records Guarantee and the local Caldicott guardian. Ultimately, a series of pragmatic solutions were reached, for example, by issuing primary care trust analysts with honorary contracts at each participating general practice.

A notable feature of the Devon virtual ward was that staff had admitting privileges at South Molton community hospital and had access to the general practice electronic health record. Indeed, to help with the process of integration, an electronic single assessment process and common assessment framework were introduced [21]. However, these assessment systems proved to be too unwieldy for practical use. A more successful initiative was the virtual ward data exchange, which enabled the sharing of information across organisational boundaries by bringing together data from the acute hospitals, general practitioner systems, out-of-hours providers, the local ambulance trust, as well as social care and community health services. For the first time, it became possible to obtain a ‘whole system’ view of each individual patient's data. This system was seen as a very powerful tool for promoting integration, not least because it allowed whole-system costs to be determined for each member of the population.

#### Multidisciplinary teams

The Devon virtual ward project was staffed by a multidisciplinary team drawn from several health and social care organisations. Over the course of the project, additional team members were added, including discharge coordinators from the local acute trust, representatives of the British Red Cross *Home from Hospital* service, and a pharmacist.

As the project developed, members of the virtual ward teams were granted increased access to different organisations’ information technology infrastructure. For example, real-time synchronisation was established between the general practice electronic record in some practices and the electronic records system used by community health services, social care teams and the out-of-hours service. To facilitate this integration, a range of common standards was first agreed, which covered issues such as data confidentiality and care coordination.

#### Activated patients

The degree to which different virtual ward patients were actively involved in their own health care varied considerably. Where appropriate, virtual ward patients were referred by virtual ward staff to the *Expert Patients Programme* [22], or they were offered cognitive behavioural therapy. However, although virtual ward patients were actively encouraged to participate in care planning, in reality the uptake was often low.

### Wandsworth

The most notable feature of the Wandsworth virtual wards project was the inclusion of a full-time general practitioner as part of the virtual ward team. This new role for general practitioners was viewed as the primary care equivalent of intensive care doctors in a hospital, in the sense that they dealt with only the most complex patients within a given population.

As in Devon, there was a clear general practitioner champion supporting the virtual ward project in Wandsworth. In comparison with the other two study sites, social care colleagues were much more closely involved. Another difference was that the virtual wards in Wandsworth used the *Patients at Risk of Re-hospitalisation* model [5] to identify high-risk patients, in contrast to Devon and Croydon, which used the Combined Predictive Model. The patients at risk of re-hospitalisation model is limited to looking at people who have had a prior hospital admission, and so it identifies fewer high-risk patients than the Combined Predictive Model, which looks at whole populations. As a result, the Wandsworth virtual wards also accepted clinical referrals to increase the number of patients - an arrangement that was popular among local general practitioners, who were able to refer their ‘difficult-to-manage’ patients. Ultimately, only about one quarter of the patients in Wandsworth were identified using a predictive model, with the remainder being clinical referrals.

#### Operating environment

Virtual wards were not established in Wandsworth as a direct response to any specific national or primary care trust policy; rather, this was a general practice-led initiative based on conversations with the established virtual ward teams in the neighbouring London borough of Croydon. Being general practice-led, the project in Wandsworth remained relatively free from influence and regulation by the primary care trust and there was nothing in the broader operating environment that directly undermined the project.

The initial start-up costs for the project were paid by Wandsworth Primary Care Trust, which also funded a project manager. The virtual ward general practitioners were salaried employees of the primary care trust until the local acute trust took over their contract, at which time they became employees of the acute trust.

#### Organisational culture, infrastructure and processes

At the start of the project, there were very few cross-organisational processes in place: for example, there was no shared information technology**. There was, however, a general appetite for better horizontal integration between health and social care, and vertical integration between primary, community and hospital care. As the project progressed, primary care trust managers became increasingly supportive. Indeed, the integrative processes developed in the virtual wards came to be regarded as trailblazing.

As with Croydon and Devon, the Wandsworth virtual wards were not supported by a fully integrated information technology system. Virtual ward staff used the general practice electronic record for recording their notes rather than using the community services’ information technology system. However, staff did also have the ability to access the information technology systems used by colleagues working in social services, community services and secondary care.

Over time, the virtual ward team began using shared assessments and developed shared care plans; however, social services staff and mental health staff continued to use separate assessments. Overall, the implementation of common standards was hampered by cross-organisational cultural differences. A prime example related to means-testing for social care services, a practice that was seen by health service staff as conflicting with the National Health Service's ethos of providing care to all that is free at the point of delivery.

#### Multidisciplinary teams

In Wandsworth, the virtual wards (which were subsequently renamed ‘community wards’) consisted of multidisciplinary teams with members drawn from several organisations, including the primary care trust and the local authority. Due to recruitment difficulties, staffing levels did not reach their full capacity in any of the four virtual wards for some considerable time. Nonetheless, practitioners working in the virtual wards were generally positive about the virtual ward concept, although they were sometimes resistant at first to change their own working patterns to accommodate this new way of working. In other words, the default was to work *reactively* as an individual practitioner rather than *proactively* as part of a multidisciplinary team.

#### Activated patients

In Wandsworth, the process of ‘activating’ patients to take a more active role in their health care was seen as one of the key roles of the virtual ward.

## Discussion

Any analysis based on case studies is going to face the challenge of producing findings that are generalisable. However, we believe that it is important to document the nature of integrated care interventions, especially at a time when new organisational models for managing chronic disease are emerging all the time, and when the labels that are applied to these ‘innovations’ are neither consistent nor always clear about the nature of the service offered.

The principal goal of the virtual wards in Devon, Croydon and Wandsworth was to reduce rates of unplanned hospital admissions and readmissions in the local population. Since patients at high risk of hospitalisation frequently have complex health and social care needs, such patients are typically cared for by several practitioners belonging to more than one health and social care team. This complexity often leads to fragmentation in care, and also to failures of communication, which may manifest themselves as gaps in care or as unnecessary duplications in care (i.e. where one professional mistakenly assumes that another professional has or has not addressed a clinical issue, respectively). Since these patients have multifaceted needs, they tend to be ill served by single-disease protocols and care pathways. Instead, the organising principle for care integration in these virtual ward projects was to make the individual patient the focus of care.

In all three study sites, organisational support for the goals of the project was reinforced by policies aimed at reducing rates of unplanned hospital admissions and at improving the quality and cost effectiveness of the care provided to patients with long-term conditions. In two of the case studies, there was a ‘general practitioner champion’ who assumed overall responsibility for the project; however, there was no equivalent sponsor in Croydon beyond an initial pilot. It seems that having a general practice sponsor was invaluable for engaging the general practice community and it also appears to have helped sustain the multidisciplinary nature of the intervention in Devon and Wandsworth. Moreover, the inclusion of specific funding for risk stratification and case management as part of the general practice contract (known as a *directed enhanced service*) is likely to ensure continued primary care support for this model of care in these two sites.

It is important to recognise that virtual wards were established at the same time as several other improvement programmes were being implemented in England. In Croydon, for example, the virtual ward project coincided with the creation of the community matron role and the launch of a national policy to provide life-long case management to high-risk patients [23]. Similarly, in Devon and Wandsworth, the virtual wards were launched at the same time that the use of telemonitoring devices was being promoted [24] and the establishment of *Partnerships for Older People Pilots* [25]. The fact that all of these programmes were being implemented concurrently has added to the complexity of integrated care in all of the study sites.

Some of the most important integrative process in the virtual ward project involved pooling data from primary care and secondary care to run the Combined Predictive Model [13]. Other notable integrative processes included agreeing with partner organisations which patients would be eligible for the service and holding regular multidisciplinary team meetings (‘ward rounds’) attended by professionals from community health care, primary care and social care. These joint clinical meetings helped foster a set of shared values, even where the financial incentives and administrative processes were not fully aligned.

Data sharing and information management were instrumental to all three projects: in Croydon and in Devon, Read code data from general practice clinical systems were used to run the predictive models; and in Wandsworth and Devon, members of the virtual ward clinical team were able to read and write in the general practice electronic record.

Across the three sites, integration happened mostly at the micro-level (i.e. patients’ interactions with different professionals and clinical teams) and at the meso-level (i.e. clinical structures and processes) [26]. Specifically, integration resulted from the focus that virtual wards provided (i.e. focusing on a restricted number of high-risk patients for whom the virtual ward team had a shared responsibility), and by providing a forum in which the care of these patients could be discussed (i.e. regular multidisciplinary team meetings).

As we have seen, the financial and policy motivations for integration varied across the different professional groups, teams and organisations that were involved in the commissioning and provision of virtual ward care in the three sites. More recently, some of the previously misaligned financial incentives have started to be addressed. For example, since April 2011, National Health Service hospitals have been incentivised to reduce their 30-day readmission rates and to cap their total number of admissions in a year [27]. This means that hospitals are now taking more interest in hospital avoidance initiatives such as virtual wards, especially in sites where the local acute trust is not the provider of community health services. Another innovation is the *Year-of-Care* funding model - a form of capitation that may also promote better integration in vertically integrated trusts [28].

Within the virtual wards projects, integration occurred mostly at the coordination and linkage levels, rather than through full financial and organisational integration [18]. It seems that this degree of integration was probably appropriate, given the project's focused goal of preventing hospitalisation in a limited number of patients. However, it is important to remember that patients at very high-predicted risk of hospitalisation only account for a modest proportion of all unplanned admissions. Therefore, to have a meaningful impact on admission rates at the population level, it will be important to consider less-intensive, lower-cost interventions for patients at moderately high-predicted risk [29]. Indeed, there is a danger that by focusing exclusively on the integration of care for very high-risk patients, virtual wards may be diverting attention away from the integration of care for lower risk patients.

## Conclusion

The virtual ward projects in Croydon, Devon and Wandsworth represent a novel approach to integrating care for patients at high risk of unplanned hospital admission. High-risk patients often experience fragmented care; therefore, this is a fertile area for improving integration. Many high-income countries regard reducing unplanned hospitalisation rates as a strategy for improving the cost effectiveness and quality of care for their populations and for moving care away from hospitals and into the home. Virtual wards could provide both a focus for integration (i.e. a small, defined population) and a forum at which that integration can occur (i.e. regular multidisciplinary meetings or ‘ward rounds’). However, the experience in Croydon, where multidisciplinary care gave way to one-to-one case management, is a reminder of the challenges of maintaining new work patterns.

These case studies suggest a number of lessons for policy-makers. First is the importance of involving general practitioners in the design and delivery of innovative models of care: simply obtaining the backing of a general practice-led committee may be insufficient. Second, that safeguards, such as *key performance indicators*, may be helpful in avoiding regression back to old ways of working (e.g. record the number of professions represented at each multidisciplinary team meeting). Third, that an assessment of the efficacy of virtual wards in reducing unplanned hospital admissions cannot be made on the pooled results of these three case studies, since the nature of the intervention varies so widely. Finally, that there may be advantages to greater standardisation across projects, both in order to facilitate evaluation with sufficient numbers of patients and homogeneity of the intervention, but also to safeguard fidelity to the model itself. For example, in the USA the GRACE project [30] is also a model of multidisciplinary case management but it sets out very detailed specifications including the timing of patient reviews, follow-up calls and visits to patients’ homes.

## Figures and Tables

**Box 1.**
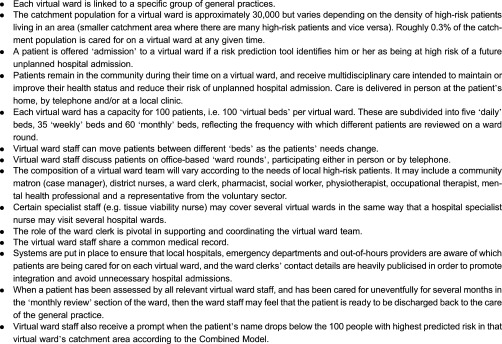
‘Original’ model of virtual wards [6].

**Table 1.**
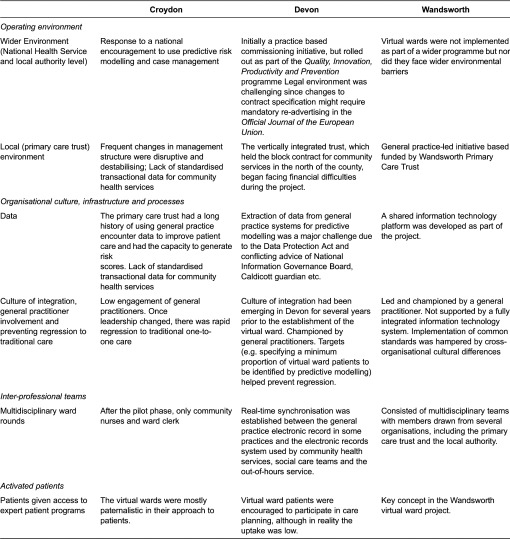
Overview of the virtual ward intervention in each study site [9]

**Table 2.**
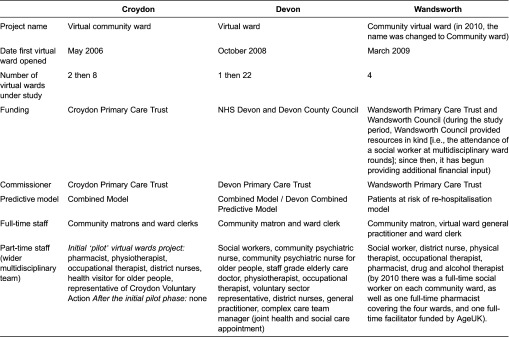
Summary observations on the attributes of the three pilot sites
